# Comparative *in vitro* activity of ciprofloxacin and levofloxacin against isolated uropathogens in Ghana: a pilot study

**DOI:** 10.11604/pamj.2018.30.194.15457

**Published:** 2018-07-05

**Authors:** Daniel Kwame Afriyie, Linda Brakowaah Adu, Marc Dzradosi, Seth Kwabena Amponsah, Prince Ohene-Manu, Francis Manu-Ofei

**Affiliations:** 1 Ghana Police Hospital, Pharmacy Department, Accra, Ghana; 2 Department of Pharmacology and Clinical Pharmacy, School of Pharmacy, Central University, Accra, Ghana; 3 Department of Pharmacology and Toxicology, School of Pharmacy, University of Ghana, Accra, Ghana

**Keywords:** Fluoroquinolones, activity, uropathogens, susceptibility

## Abstract

**Introduction:**

Studies on urinary tract infections (UTIs) in West Africa from 1990 to 2012 have showed moderate to high antimicrobial resistance to commonly prescribed antibiotics. Fluoroquinolones have been the main stay in the management of UTIs, but recent reports show emergence of resistance. Levofloxacin and ciprofloxacin still remain the commonest fluoroquinolones prescribed for UTIs in many settings. objective: this study sought to compare activity of ciprofloxacin and levofloxacin against clinical isolates obtained from patients with suspected UTI at the Ghana Police Hospital.

**Methods:**

Midstream urine samples from 153 suspected UTI patients who visited the Ghana Police Hospital from July 2016 to March 2017 were examined. Urine samples were cultured and isolates identified by standard biochemical and serological methods. The Kirby-Bauer disc diffusion method was used to determine susceptibility of isolates to ciprofloxacin and levofloxacin.

**Results:**

UTI prevalence was significantly (p < 0.05) higher among female patients (74.5%) than male patients (25.5%). Clinical uropathogens isolated from urine samples were *Escherichia coli* (28.1%), *Coliform spp* (43.2%), *Klebsiella spp* (26.1%) and *Staphylococcus aureus* (2.6%). Overall sensitivity of the uropathogens to ciprofloxacin and levofloxacin were 77.1% and 62.8%, respectively. *Staphylococcus aureus*showed greater resistance to levofloxacin (75%) compared to ciprofloxacin (25%). All Gram-negative isolates showed a higher sensitivity to ciprofloxacin compared to levofloxacin: *Escherichia coli*; 69.8% vrs 62.8%, *Coliform sp*p; 80.3% vrs 65.2%, and *Klebsiella spp*; 80% vrs 62.5%.

**Conclusion:**

This study revealed emergence of resistance of uropathogens to quinolones. The isolates showed higher sensitivity to ciprofloxacin compared to levofloxacin. Rational prescribing and use of these fluoroquinolones following local susceptibility data is thus recommended.

## Introduction

Urinary tract infection (UTI) is one of the commonly reported microbial infections at healthcare settings. UTI can affect all ages and both sexes, although prevalence is known to be higher amongst females [1]. Reports suggest that there could be spontaneous resolution of UTI (rates between 50 - 70%) without any pharmacologic intervention [2]. Effective management of UTI would usually require the use of antimicrobial agents. However, reports show increasing resistance among certain urinary pathogens (uropathogens) to some commonly used antimicrobial agents. A number of studies report resistance of uropathogens to some first line antibiotic therapy: 10 - 20% for trimethoprim and sulfamethoxazole (TMP-SMX), 40% for ampicillin, 15 - 20% for nitrofurantoin and less than 10% for the fluoroquinolones [2-5]. Among the various antimicrobials used in the treatment of UTIs, the fluoroquinolones remain the preferred class. This is due to the fact that fluoroquinolones have high bacteriologic and clinical cure rates, low resistance rates, and few adverse drug reactions [6,7]. Fluoroquinolones are bactericidal antibiotics that target specifically DNA gyrase of the microorganism. Amongst the most frequently prescribed fluoroquinolones for empirical treatment of UTIs are ciprofloxacin and levofloxacin [3, 8, 9]. Ciprofloxacin, a second-generation fluoroquinolone, has greater activity against Gram-negative bacteria compared to nalidixic acid (a first-generation quinolone). Levofloxacin, a third-generation fluoroquinolone, is known to have broad spectrum of activity against Gram-positive and Gram-negative bacteria, and atypical pathogens [10]. Despite the preference for fluoroquinolones in the management of UTIs, there are current reports that suggest emergence of uropathogen resistance to commonly prescribed fluoroquinolones [11,12]. Data from Ghana and elsewhere show that isolated uropathogens have higher resistance to ciprofloxacin compared to levofloxacin [13-15]. As a result of this, healthcare professionals in Ghana may sometimes prescribe levofloxacin instead of ciprofloxacin (which is first-line treatment)for uncomplicated UTI. Two recent studies at a secondary level quasi-government hospital in Ghana showed that isolated uropathogen exhibited 35.9 % resistance to ciprofloxacin [16] and 51.6% resistance to levofloxacin [17]. Data from the latter study showed that levofloxacin resistance among uropathogens was on the rise. Therefore, the current study sought to compare the susceptibility of uropathogens (clinical isolates) to levofloxacin and ciprofloxacin concurrently.

## Methods

**Study site**: The study was conducted at a secondary level quasi-government hospital, Ghana Police Hospital. The Hospital is a 100-bed facility providing healthcare to police personnel, their dependents, and the general public. The Out-patient Department of the Hospital provides service to over 100,000 patients each year.

**Study design and population**: This was a cross-sectional study that screened urine samples collected from suspected UTI patients (both In-and Out-patients) who visited this secondary level quasi-government hospital from July 2016 to March 2017.

**Urine processing**: Midstream urine samples from patients were collected into sterile urine containers, stored between 2^o^ - 4^o^C in a refrigerator, and analyzed not later than 4 hours. All analysis were done at the Microbiology Unit of the Ghana Police Hospital. Culture of urine was done with the aid of a calibrated wire. A loop-full (0.01 mL) of each sample of urine was inoculated onto a quarter-plate of Cysteine Lactose Electrolyte-Deficient (CLED) agar (Biotic Laboratories Ltd, UK). Specimen were streaked on agar plates to allow discrete colonies and incubated at 37^o^C for 24 hours in Nodermann GMBH incubator (Germany). After the 24-hour incubation period, colonies were enumerated and those with significant growth identified. Significant microbial growth was defined as a culture of single microorganisms at a concentration ≥ 1×10^5^cfu/mL. Microbial colonies were identified using morphological features, standard biochemical and serological methods [18]. After identification, each colony, representing an isolate was emulsified in 2 mL sterile peptone water, and then transferred into sensitivity agar plates (Biotec laboratories, UK).

**Susceptibility test**: Susceptibility of isolated uropathogens to levofloxacin and ciprofloxacin was done using the Kirby-Bauer disc diffusion method. Sensitivity discs of levofloxacin (5 µg) and ciprofloxacin (5 µg) obtained from Biomark Laboratories, India (BDR001, Lot: 0215/0686) were stored under recommended refrigeration (-200 C to 80 C) until used for tests. Inhibition zone diameters pertaining to levofloxacin and ciprofloxacin were measured using calipers and compared with standard interpretation charts, and scored as sensitive or resistant.

**Statistical analysis**: Data was entered into Statistical Package for the Social Sciences (SPSS) Version 21 (IBM Corporation, USA) and checked for completeness. Thereafter, descriptive statistics (percentages and frequencies) was used to present findings. Student's t-test was used to compare difference between continuous variables. A p < 0.05 was considered statistically significant.

**Ethics**: Ethical approval for this study protocol was obtained from the Ghana Police Hospital Administration. Patients' data were handled with utmost confidentiality and all patient identifiers were removed.

## Results

Of the 153 urine samples with significant bacteria growth that were examined, 114 (74.5%) were from females and 39 (25.5%) from males. Patients of ages between 20-39 years had the highest prevalence of clinical UTI, 76 (49.7%), whilst the lowest prevalence was observed in patients less than 20 years, 21 (13.7%). Age categories with respective UTI cases are presented in Table 1. *Coliform spp* was the most predominant urinary pathogen isolated, 66 (43.2%) out of the 153 isolates. *Escherichia coli, Klebsiella spp* and *Staphylococcus aureus* (the only Gram-positive) constituted 43 (28.1%), 40 (26.1%) and 4 (2.6%) respectively, of the total isolates. The overall sensitivity of isolated uropathogens to ciprofloxacin was 77.1%, and levofloxacin 62.8%. The only Gram-positive isolate *Staphylococcus aureus* was more susceptible to ciprofloxacin (75%) compared to levofloxacin (25%). On the whole, the Gram-negative isolates (*Escherichia coli, Klebsiella spp* and *Staphylococcus aureus*) were more susceptible to ciprofloxacin compared to levofloxacin, as shown in Table 2.

**Table 1:** Age and sex distribution of patients with UTIs at the quasi-government hospital

GENDER	˂20 YEARS	20-39YEARS	40-59YEARS	≥60YEARS	TOTAL
Females	14	61	27	12	114 (74.5%)
Males	7	15	6	11	39 (25.5%)
Total	21 (13.7%)	76 (49.7%)	33 (21.6%)	23 (15.0%)	153 (100%)

**Table 2:** Susceptibility pattern of isolated uropathogens to ciprofloxacin and levofloxacin

	CIPROFLOXACIN		LEVOFLOXACIN	
ISOLATES	Sensitivity	Resistance	Sensitivity	Resistance
*Escherichia coli* (n = 43)	30 (69.8%)	13 (30.2%)	27 (62.8%)	16 (37.2%)
*Coliform spp* (n = 66)	53 (80.3%)	13 (20.7%)	43 (65.2%)	23 (34.8%)
*Klebsiella spp* (n = 40)	32 (80.0%)	8 (20.0%)	25 (62.5%)	15 (37.5%)
*Staphylococcus aureus* (n = 4)	3 (75.0%)	1 (25.0%)	1 (25.0%)	3 (75.0%)
**Overall Sensitivity**	**118 (77.1%)**	**35 (22.9%)**	**96 (62.8%)**	**57 (37.2%)**

## Discussion

Regular laboratory-based surveillance is important for local and national action in the monitoring of antimicrobial resistance (AMR) and its spread [19,20]. Studies of such nature promote rational prescribing of antibiotics, and provide up-to-date information that direct policies aimed at addressing AMR. The current study showed that the prevalence of UTI was significantly (p < 0.05) higher in females (74.5%) compared to males (25.5%).This corroborates a study in Nigeria, where the prevalence of UTI was higher among females, 55.1%, than in males, 34.9% [21]. Other studies elsewhere have also showed that the prevalence of UTI in females is higher than in males [22,23]. Factors such as hormonal changes during pregnancy, anatomical difference between the male and female urethra, proximity of the male and female urethra to anal opening, poor personal hygiene, some cultural practices among women and the absence of prostatic secretion in females could be underlying factors for the moderately high UTI prevalence observed among females than their male counterparts [23]. The age range with the highest infection, 49.7%, was in patient within 20-39 years. Those with ages less than 20 years had the lowest infection (13.7%). The high rate of UTI among the age category 20-39 years could be due to the high incidence of sexual activity and/or multiple sex partners known to be associated with that age group [24]. UTI among patients within the age range of 40-59 (12.6%) and above 60 years (15%) observed in this study could be due to factors such as prostatic enlargement in males, reduced ambulation, catheterization, diabetes mellitus and weak bladder sphincters [25]. Studies conducted in Ghana have shown that, *Escherichia coli, Klebsiella, Candida* and *Salmonella* speciesare among the commonest isolated uropathogens from patients with UTI [26, 27]. Reports from similar studies elsewhere revealed that, common pathogens in laboratory cultures of urine are *Escherichia coli* and *Enterobacteriaceae* [28, 29]. Previous studies conducted at the Ghana Police Hospital reported the presence of fungi, mixed fungi and bacteria, and mixed bacteria isolates [16,17]. In the current study, bacteria were the pathogens isolated; *Coliforms spp, Escherichia coli, Klebsiella spp* and *Staphylococcus aureus*. The current study also showed that *Escherichia coli* and *Coliform spp* were the most prevalent uropathogens, accounting for 71.3% of the clinical isolates. Similar studies in Ghana; Kumasi and Cape Coast Hospitals, however, reported *Escherichia coli* and *Klebsiella spp* as the most prevalent bacteria isolates responsible for UTI [30, 31].

Although fluoroquinolones have been useful in the treatment of UTI, current reports in a number of countries, Ghana inclusive, suggest an increase in microbial resistance [13,14,32,33]. A possible reason for this could be indiscriminate use of fluoroquinolones in the treatment of human and animal infections [34]. The current study revealed that, overall susceptibility of microbial organisms to levofloxacin was 62.8% and to ciprofloxacin was 77.1%. This observation suggests that, isolated uropathogens were more susceptible to ciprofloxacin than levofloxacin. On the contrary, findings from a similar study in a tertiary-care hospital in India reported uropathogen susceptibility of 46.2% for levofloxacin and 14.5% for ciprofloxacin [35]. Resistance of isolated uropathogens to ciprofloxacin and levofloxacin in this study was 23.9% and 37.2% respectively. The level of resistance to these two fluoroquinolones were higher than the 10% resistance observed in a similar study in United States of America [5], but lower than findings from the study in India [35]. Further analysis of results also showed that, resistance of levofloxacin and ciprofloxacin to uropathogens in this study was lower than fluoroquinolone resistance (> 50%) reported after a recent nationwide antimicrobial resistance surveillance in Ghana [33]. Findings from this study (emerging resistance to fluoroquinolones) tend to agree with conclusions from a systematic review and meta-analysis of UTIs in West Africa from 1990 to 2012 by Bernabe et al [36]. Several studies have revealed that levofloxacin has better activity against Gram-positive bacteria, and less likely to select for resistant strains compared to the older quinolones [6,37,38]. However, the only Gram-positive isolate *Staphylococcus aureus*, identified in this study showed higher (75%) resistance to levofloxacin compared to ciprofloxacin (25%). This observation is contrary to the fact that third generation fluoroquinolones such as levofloxacin possess broad spectrum of activity against Gram-positive and Gram-negative bacteria than ciprofloxacin (second generation fluoroquinolone). Further analysis of data revealed that ciprofloxacin had greater activity against Gram-negative bacteria isolates; *Coliforms spp, Escherichia coli* and *Klebsiella spp*, than levofloxacin. Thus, suggesting ciprofloxacin has greater activity compared to levofloxacin. A limitation of this study was the fact that the study was conducted in only one health facility; hence findings may not be the reflection of what pertains elsewhere. Another limitation was the small sample size of the study (n = 153). This was due to the limited number of levofloxacin and ciprofloxacin discs available for the study.

## Conclusion

In conclusion, this study showed that among uropathogens isolated at this quasi-government hospital, ciprofloxacin had greater activity than levofloxacin. The high resistance of isolated uropathogens to the relatively new levofloxacin (a third generation fluoroquinolone) observed in this study is noteworthy. Results from this study re-emphasize the need for rational use of fluoroquinolones during empirical treatment of UTIs, and/or the need for antimicrobial sensitivity tests before drug treatment. Furthermore, findings from this research provide a basis for a multicenter study. This would enhance rational, evidenced-based, and cost-effective use of these antibiotics in UTIs management in Ghana and elsewhere.

### What is known about this topic

- UTIs is a common microbial infection which affects all ages and sexes globally;
- There is increased microbial resistance to commonly prescribed antibiotics, including the quinolones;
- Need for regular antimicrobial resistance surveillance to guide prudent treatment.

### What this study adds

- Despite ciprofloxacin being an older generation fluoroquinolone, it appears to remain the drug of choice in empiric treatment of uncomplicated UTI as against the relatively newer third generation levofloxacin;
- Findings from this study that have shown the efficacy of ciprofloxacin in UTI management and provides data to further support current National Treatment Guidelines (2017) recommendation for its use in treating both uncomplicated and complicated UTIs.

## Competing interests

The authors declare no competing interests.
